# [Retracted] Resistance to the mTOR inhibitor everolimus is reversed by the downregulation of survivin in breast cancer cells

**DOI:** 10.3892/ol.2023.13938

**Published:** 2023-07-03

**Authors:** Ludovica Taglieri, Francesca De Iuliis, Anna Giuffrida, Sabrina Giantulli, Ida Silvestri, Susanna Scarpa

Oncol Lett 14: 3832–3838, 2017; DOI: 10.3892/ol.2017.6597

Following the publication of this paper, it was drawn to the Editor's attention by a concerned reader that the β-actin western blot control data shown in Fig. 2A and B on p. 3836 were strikingly similar, even though the images appeared to have been horizontally flipped comparing between the two figure parts. In addition, certain of the caspase-8 data shown in Fig. 1D on p. 3825 and in Fig. 2B appeared strikingly to be similar, again with the apparent horizontal flipping of the bands shown in adjacent lanes within Fig. 1D itself.

After having conducted an independent investigation of these figures in the Editorial Office, the Editor of *Oncology Letters* has decided that this paper should be retracted from the Journal on account of a lack of confidence in the presented data. The authors were asked for an explanation to account for these concerns, but the Editorial Office did not receive a satisfactory reply. The Editor apologizes to the readership for any inconvenience caused.

